# Study protocol: safety correction of high dose antipsychotic polypharmacy in Japan

**DOI:** 10.1186/1471-244X-14-103

**Published:** 2014-04-07

**Authors:** Tsuruhei Sukegawa, Ataru Inagaki, Yoshio Yamanouchi, Toshiya Inada, Takashi Yoshio, Reiji Yoshimura, Nakao Iwata

**Affiliations:** 1 National Hospital Organization, Tottori Medical Center, 876, Mitsu, Tottori-shi, Tottori 689-0203, Japan; 2 School of International Politics, Economics and Communication, Aoyama Gakuin University, 4-4-25, Shibuya, Shibuya-ku, Tokyo 150-8366, Japan; 3 Department of Social Psychiatry, National Institute of Mental Health, National Center of Neurology and Psychiatry, 4-1-1, Ogawahigashi-cho, Kodaira, Tokyo 187-8551, Japan; 4 Seiwa Hospital, Institute of Neuropsychiatry, 91, Benten-cho, Shinjuku-ku, Tokyo 162-0851, Japan; 5 Department of Clinical Pharmacy Faculty of Pharmaceutical Sciences, Toho University, 2-2-1, MiyamaFunabashi, Chiba 274-8510, Japan; 6 Department of Psychiatry, University of Occupational and Environmental Health, 1-1, Iseigaoka, Hachimannishi-ku, Kitakyushu, Fukuoka 807-0804, Japan; 7 Department of Psychiatry, Fujita Health University School of Medicine, Denrakugakubo, Kutsukake-cho, Toyoake, Aichi 470-1101, Japan

**Keywords:** Schizophrenia, Antipsychotics, Polypharmacy, High dose

## Abstract

**Background:**

In Japan, combination therapy with high doses of antipsychotic drugs is common, but as a consequence, many patients with schizophrenia report extrapyramidal and autonomic nervous system side effects. To resolve this, we proposed a method of safety correction of high dose antipsychotic polypharmacy (the SCAP method), in which the initial total dose of all antipsychotic drugs is calculated and converted to a chlorpromazine equivalent (expressed as milligrams of chlorpromazine, mg CP). The doses of low-potency antipsychotic drugs are then reduced by ≤ 25 mg CP/week, and the doses of high-potency antipsychotics are decreased at a rate of ≤50 mg CP/week. Although a randomized, case-controlled comparative study has demonstrated the safety of this method, the number of participants was relatively small and its results required further validation. In this study of the SCAP method, we aimed to substantially increase the number of participants.

**Methods/design:**

The participants were in- or outpatients treated with two or more antipsychotics at doses of 500–1,500 mg CP/day. Consenting participants were randomized into control and dose reduction groups. In the control group, patients continued with their normal regimen for 3 months without a dose change before undergoing the SCAP protocol. The dose reduction group followed the SCAP strategy over 3–6 months with a subsequent 3-month follow-up period. Outcome measures were measured at baseline and then at 3-month intervals, and included clinical symptoms measured on the Manchester scale, the extent of extrapyramidal and autonomic side effects, and quality of life using the Euro QOL scale. We also measured blood drug concentrations and drug efficacy-associated biochemical parameters. The Brief Assessment of Cognition in Schizophrenia, Japanese version, was also undertaken in centers where it was available.

**Discussion:**

The safety and efficacy of the SCAP method required further validation in a large randomized trial. The design of this study aimed to address some of the limitations of the previous case-controlled study, to build a more robust evidence base to assist clinicians in their efforts to reduce potentially harmful polypharmacy in this vulnerable group of patients.

**Trial registration:**

UMIN Clinical Trials Registry 000004511.

## Background

Antipsychotics should be used as monotherapy, but in many countries patients may be prescribed two or more antipsychotic drugs as combination therapy [1]. The use of more than one antipsychotic drug is particularly prevalent in East Asia [2], and polypharmacy with high dose antipsychotics is relatively common in Japan [3].

As antipsychotic polypharmacy may cause adverse drug reactions [4], these drugs should be used as monotherapy at the minimum necessary dose whenever possible. After a warning on the inappropriate use of antipsychotics was issued in Japan, the situation improved to some extent [5]. Nonetheless, dose reduction may be challenging in patients receiving high dose antipsychotic polypharmacy. We have previously proposed a protocol for the gradual reduction of antipsychotic drug dose for patients on high doses of more than one agent [6].

We examined the safety and efficacy of this method in a randomized, case-controlled comparative study (the Reduction and Simplification [RAS] study) [7]; however, the number of participants was small and the results required further validation. To support the reliability of this method, we conducted a randomized, case-controlled comparative study (the “Safety Correction of high dose Antipsychotic Polypharmacy [SCAP] study). This article describes the methods used.

### The SCAP protocol

The chlorpromazine equivalent dose (mg) is expressed as “mg CP”. To convert the doses of various antipsychotics to chlorpromazine equivalent doses, we used the calculation table prepared by Inagaki and colleagues, which is routinely adopted in Japan [8]. Antipsychotics of which the doses equivalent to 100 mg of chlorpromazine are 10 mg or less were regarded as high-potency drugs, and those of which the doses equivalent to 100 mg of chlorpromazine exceed 10 mg were regarded as low-potency drugs.

Tanabe [9] and Murasugi et al. [10] have published articles reporting both successful and unsuccessful strategies for reducing the doses of antipsychotics, including the extent of dose reduction reported as a chlorpromazine equivalent dose, and the dose reduction period. On the basis of these studies, we calculated the rate of dose reduction in those that succeeded in reducing the dose and those who failed. In participants who failed to reduce the dose, the rate of reduction was approximately 100 mg CP per week in patients initially taking 2,000 mg CP daily (Table 1). In those in whom the dose was reduced successfully, the rate of reduction was about 40 mg CP per week in patients in whom the starting dose was 1,400 mg CP. As such, the maximum acceptable reduction speed may be 50 mg CP or less per week. Low-potency antipsychotics’ different affinity for dopaminergic D_2_ receptors, acetylcholine receptors, histamine receptors and serotonin receptors compared with high-potency antipsychotics require doses 10–100 times higher than those of high-potency antipsychotics. When decreasing the doses of drugs with potent anticholinergic actions, anticholinergic withdrawal symptoms such as insomnia, anxiety and restlessness may occur. To take this into account, the rate of reduction of low-potency antipsychotics was established as being half that of high-potency antipsychotics, namely 25 mg or less per week. Furthermore, it was established that the total amount of drug that can be subtracted from the daily dose, that is the maximum acceptable amount of reduction, was twice the value of the maximum acceptable reduction rate. This amount was applied to each drug, and the actual dose of the drug (not a chlorpromazine equivalent dose) was calculated as shown in Tables 2 and 3[11]. The SCAP study was conducted in accordance with the maximum acceptable amount of dose reduction and maximum acceptable reduction rate based on these tables.

**Table 1:** Rates of reduction used in previous research

Tanabe’s research [9]					
Group	Mean dose before reduction	Reduction speed	GAF	SOI	n
Successful	1,372 mg CP	40.4 mg CP/week	34.1	4.5	37
Unsuccessful	1,832 mg CP	95.4 mg CP/week	29.8	4.8	11
Murasugi’s research [10]					
Group	Mean dose before reduction	Reduction speed	GAF	SOI	n
Successful	1,581 mg CP	43.0 mg CP/week	35.2	4.8	5
Unsuccessful	2,389 mg CP	97.4 mg CP/week	44.0	4.5	5
Combination of both studies					
Group	Mean dose before reduction	Reduction speed	GAF	SOI	n
Successful	1,397 mg CP	40.7 mg/week	33.5	4.5	42
Unsuccessful	2, 006 mgCP	96.0 mg/week	34.2	4.7	16

*Abbreviations*: *GAF* global assessment of functioning in DSM-4-TR; *SOI* severity of illness; n number of patients.

**Table 2:** Reduction protocol for high-potency drugs

**Generic name**	**Product name**	**Maximum reduction rate**^**†**^**(mg/week)**	**Maximum reduction**^**‡**^**(mg)**
Perphenazine	PZC, others	5	10
Perospirone	Lullan	4	8
Trifluoperazine	Trifluoperazine	2.5	5
Nemonapride	Emilace	2.25	4.5
Aripiprazole	Abilify	2	4
Blonanserin	Lonasen	2	4
Pimozide	OLAP	2	4
Olanzapine	Zyprexa	1.25	2.5
Bromperidol	Impromen	1	2
Haloperidol	Serenace, others	1	2
Fluphenazine	Flumezin	1	2
Timiperone	Tolopelone	0.65	1.3
Spiperone	Spiropitan	0.5	1
Risperidone	Risperdal, others	0.5	1

^†^Maximum reduction rate represents the maximum acceptable reduction per week.

^‡^Maximum reduction represents the maximum amount of reduction at a time. After maximum reduction, subsequent reduction is performed after a minimum 2-week observation period.

(Cited and partially modified from Sukegawa [11]).

**Table 3:** Reduction protocol for low-potency drugs

**Generic name**	**Product name**	**Maximum reduction rate**^**† **^**(mg/week)**	**Maximum reduction**^**‡ **^**(mg)**
Sulpiride	Dogmatyl, others	50	100
Sultopride	Barnetil	50	100
Pipamperone	Propitan	50	100
Chlorpromazine	Contomin, others	25	50
Levomepromazine	Levotomin, others	25	50
Carpiprammine	Defecton	25	50
Oxypertine	Forit	20	40
Zotepine	Lodopin, others	16.5	33
Quetiapine	Seroquel	16.5	33
Clocapramine	Clofekton	10	20
Mosapramine	Cremin	8.25	16.5
Propericiazine	Neuleptil	5	10
Prochlorperazine	Novamin	3.75	7.5
Moperone	Luvatren	3.125	6.25

^†^Maximum reduction rate represents the maximum acceptable reduction per week.

^‡^Maximum reduction represents the maximum amount of reduction at a time. After maximum reduction, subsequent reduction is performed after a minimum 2-week observation period.

(Cited and partially modified from Sukegawa [11]).

The chlorpromazine equivalent dose was adopted as a means of calculating the rate of dose reduction for the following reasons. It is known that the potency of antipsychotics is proportional to their binding capacity to dopamine D_2_ receptors. In patients who have taken very high dose antipsychotics over a long period, D_2_ receptor expression may have been upregulated to compensate for D_2_ antagonism. Recurrent psychiatric symptoms induced by decreasing the doses of antipsychotics are sometimes called “dopamine supersensitivity psychosis” [12]. Therefore, we adopted the chlorpromazine equivalent dose, which represents the potency of the antipsychotic, to allow us to evaluate the degree of reduction in anti-D_2_activity caused by dose reduction.

The rate of reduction was expressed as an absolute value, not as a proportion of the total chlorpromazine equivalent dose of the antipsychotic. Intuitively, a method of decreasing the dose by a small proportion (for example 1%) of the total dose per week may be appropriate; however, when the total chlorpromazine equivalent dose of antipsychotics is high, D_2_ receptors may become supersensitive to compensate for the D_2_-mediated actions inhibited by the antipsychotics. Therefore, when decreasing the dose of an antipsychotic, dopamine supersensitivity psychosis may occur if the chlorpromazine equivalent dose of the antipsychotic administered at the start of dose reduction is high. When the dose is higher, the dose must be weaned at a reduced rate. To achieve this, the reduction speed should be considered as an absolute value, not as a proportion of the total dose.

Gradually reducing the dose of antipsychotics in this way may be a useful strategy for rationalizing high dose antipsychotic regimes. A pilot randomized study involving 39 patients at 10 institutions was completed in 2007 (the RAS study) [7]. The participants were patients with schizophrenia who had been hospitalized for 1 year or more, treated with three or more antipsychotics at a dose of 1,500 mg CP/day or higher, and were capable of understanding the explanatory document and giving informed consent. In the reduction and simplification group, the 6-month reduction and simplification protocol was completed before a 3-month follow-up period, targeting reduction by 500 mg CP per day or more and the number of antipsychotics to one or two. In the reduction and simplification group (which consisted of 19 patients taking a mean of 3.7antipsychotic drugs at a mean dose of 2,067 mg CP at the time of enrollment), the mean dose reduction was 674 mg CP, and the mean reduction in the number of drugs was 1.0. Eleven patients succeeded in reducing their dose by 500 mg CP or more. However, the number of drugs taken could be reduced to one or two in only five patients. Hallucinations or delusions recurred in four patients, which were successfully treated by dose escalation in all cases. Two patients dropped out because of transfer to another hospital or the aggravation of physical symptoms. Three patients violated the protocol, one of whom experienced recurrence of hallucinations and delusions. Overall, 11 (79%) of the 14 patients excluding those who deviated from the protocol or dropped out succeeded in reducing their dose by 500 mg or more. In the control group (which consisted of 20 patients taking a mean of 3.5antipsychotic drugs at a mean dose of 2,143 mg CP at the time of enrollment), three patients dropped out owing to the aggravation of physical symptoms, extrapyramidal adverse drug reactions, or hallucinations or delusions, and two violated the protocol. Hallucinations or delusions were reported by four of the 19 patients in the reduction and simplification group and one of the 20 patients in the control group, but this difference was not statistically significant. Comparison of the clinical characteristics of the reduction and simplification and control groups at the time of enrollment found no significant differences other than a higher proportion of women in the reduction and simplification group. After 6 and 9 months, the number of antipsychotics and chlorpromazine equivalent dose were significantly lower in the reduction and simplification group. No significant differences were noted in the evaluation or test items between the groups. When the group who achieved dose reductions of 500 mg or more (11 patients) was compared with the control group excluding those who violated the study protocol or dropped out (15 patients), the incidence of autonomic adverse drug reactions (particularly nausea and vomiting) had significantly reduced by the ninth month. A limitation of the RAS study was that there were only 39 participants. We undertook the SCAP study to confirm the usefulness of the SCAP method in a larger cohort of patients with schizophrenia.

## Methods

This was an open-label, multicenter, randomized study conducted between November 2010 and March 2012. The recurrence and dropout rates during the study period, as well as changes in psychiatric symptoms, side effects of the extrapyramidal and autonomic nervous systems, and quality of life, were compared between the dose reduction and control groups. The study was conducted in accordance with the Declaration of Helsinki and Good Clinical Practice. The study protocol was also examined and approved by the ethics review board of each center, and by the Ethics Review Board of Fujita Health University for applications from those centers without their own panel.

The participants were inpatients or outpatients, aged 20 years or older, diagnosed with schizophrenia according to the Diagnostic and Statistical Manual of Mental Disorder, 4th edition text version (DSM-IV-TR) [13], who were taking two or more antipsychotics at doses of 500–1500 mg CP/day. Written informed consent was obtained from all participants. Each participant was assigned to the dose reduction or control group according to a randomization protocol administered by Fujita Health University. A total of 163 patients were ultimately enrolled; 101 were assigned to the dose reduction group and 62 to the control group.

In the dose reduction group, the dose was decreased to 80% or less than the initial dose over 3–6 months according to the SCAP method. Participants were subsequently followed up for a further 3 months. Clinical symptoms were evaluated using the Manchester scale [14], the extent of extrapyramidal side effects using the Drug-induced Extrapyramidal Symptoms Scale (DIEPSS) [15], autonomic side effects using the Udvalg for Kliniske Undersogelser11-item scale (UKU-11) [16], and quality of life using the Euro QOL scale [17]. In addition, measurement of blood drug concentrations and drug efficacy-associated biochemical parameters were performed. The Brief Assessment of Cognition in Schizophrenia, Japanese version (BACS-J) [18] was also performed in centers where it was available.

According to the SCAP method, the doses of high-potency drugs were reduced at a rate of 50 mg CP per week or less, and those of low-potency drugs at a rate of 25 mg CP per week or less [6]. In the control group, the doses of antipsychotics were not changed for 3 months if clinically feasible, and then doses were reduced according to the same protocol.

### Sample size calculation and statistical techniques

Sample size calculation was undertaken to allow detection of non-inferiority of the dose reduction group compared with the control group on the basis of the primary outcome measure based on a binary set-up repeated measurement linear mixed model using G*power3.1.5 (http://www.gpower.hhu.de/). For repeated measures analysis of variance (ANOVA) between factors, if the α error was assumed to be 0.025 (to analyze multiple scales), statistical power was 0.8, non-inferiority margin of Cohen’s d was 0.2 (it was predicted that the difference was smaller than moderate), the correlation among repeated measures was 0.5 and the number of measurements was six, then the size of the cohort was determined as 142. On this basis, we aimed to enroll 72 participants to each group. Data were finalized on January 31, 2013. Missing values were supplemented using the mixed model repeated measures (MMRM) method and a *t*-test was performed to examine differences in the means of each outcome measure between the groups. All analyses were undertaken using the Japanese version of SPSS (version 21.0. IBM, Tokyo, Japan).

## Discussion

The doses of antipsychotics were reduced in the RAS study and the SCAP study using the same method, but the two studies are different. The RAS study had been completed by December 2007, but the SCAP study did not commence until November 2010. The main limitation of the RAS study was its small sample size, but there were additional problems with the study design. The SCAP study aimed to enroll a larger number of participants to address these limitations.

For the convenience of our investigators, we stipulated the rate of dose reduction in the SCAP study. In the RAS study, participating psychiatrists had calculated the rate of dose reduction by themselves based on SCAP criteria.

Criteria for enrollment in the SCAP study were as follows: both inpatients and outpatients were considered acceptable, treatment with two or more antipsychotics at doses of 500–1,500 mg CP, and a follow-up period of 3 months in the control group. Practically, it can be difficult to obtain written informed consent from patients taking antipsychotics at a dose in excess of 1,500 mg CP. Furthermore, some patients refuse to participate, claiming that they did not wish to be assigned to the control group and thus have to remain on an elevated dose for an extended period despite their agreement to participate in a study regarding dose reduction. We therefore established a shorter follow-up period for those in the control group, despite the fact that this might influence our findings. These elements of the SCAP study design differentiate it from the RAS study, in which only inpatients were enrolled, participants had been treated with more than three antipsychotics at doses in excess of 1,500 mg CP, and the follow-up period was 9 months in both the dose reduction and control groups.

The missing data in the control group are a consequence of our study protocol. The missing data were regarded as “Missing Completely At Random (MCAR)” or at least “Missing At Random (MAR)”. Therefore, the MMRM was thought to be most suitable for supplementation of the missing data in this study [19].

Many psychiatrists in Japan understand that very high doses of antipsychotics should be avoided, but also that overly rapid dose reduction may result in withdrawal symptoms mediated by dopaminergic D_2_, cholinergic or other receptors. These symptoms may be misdiagnosed as relapse and consequently dose reduction may be abandoned. If our findings suggest that the SCAP method is a useful means of reducing the dose of antipsychotic drugs and rationalizing antipsychotic polypharmacy, the strategy might help the prescription of antipsychotic drugs in Japan meet international standards.

## Abbreviations

SCAP: Safety correction of high-dose antipsychotic polypharmacy; RAS study: Reduction and simplification study; mg CP: mg as a chlorpromazine equivalent dose; D2 receptor: Dopamine 2 receptor; DSM-IV-TR: The diagnostic and statistical manual of mental disorders, 4th edition text version; DIEPSS: Drug-induced extrapyramidal symptoms scale; UKU-11: 11 items reporting autonomic side effects measured on the Udvalg for Kliniske Undersogelser scale; BACS-J: Brief Assessment of Cognition in Schizophrenia, Japanese version; MMRM: Mixed model repeated measures; ANOVA: Analysis of variance; MCAR: Missing completely at random; MAR: Missing at random.

## Competing interests

Competing interests are as follows: TS has received grants from Otsuka. AI and TY have received grants from the Japan Pharmaceutical Manufacturers Association. TI has received lecture fees from Otsuka, Dainippon Sumitomo, GlaxoSmithKline, Eli Lilly, Janssen, Astellas and Mitsubishi Tanabe. NI has received lecture fees from Otsuka, Janssen, GlaxoSmithKline, Shionogi and Eli Lilly, grants from Otsuka and honoraria from Otsuka, Dainippon Sumitomo, GlaxoSmithKline, Mitsubishi Tanabe, Yoshitomi yakuhin and Eizai. None of the other authors have competing interests to declare.

## Authors’ contributions

All authors conceived the study and its design, and wrote the draft manuscript. All have read and approved the final manuscript.

## Pre-publication history

The pre-publication history for this paper can be accessed here:

http://www.biomedcentral.com/1471-244X/14/103/prepub
